# Excess mortality from all causes during the COVID-19 pandemic in the city of Rio de Janeiro, Brazil

**DOI:** 10.1590/1980-549720230013

**Published:** 2023-02-20

**Authors:** Valeria Saraceni, Oswaldo Gonçalves Cruz, João Roberto Cavalcante, Fernanda Morena dos Santos Barbeiro Vieira, Bruno Baptista Cardoso, Débora Medeiros de Oliveira e Cruz, Gislani Mateus Oliveira Aguilar, Betina Durovni, Daniel Soranz, Márcio Henrique de Oliveira Garcia

**Affiliations:** I Secretaria Municipal de Saúde do Município do Rio de Janeiro – Rio de Janeiro (RJ), Brazil.; II Fundação Oswaldo Cruz – Rio de Janeiro (RJ), Brazil.; III Universidade do Estado do Rio de Janeiro – Rio de Janeiro (RJ), Brazil.

**Keywords:** COVID-19, Mortality, Epidemiology, Public health surveillance, COVID-19, Mortalidade, Epidemiologia, Vigilância em saúde pública

## Abstract

**Objective:**

To evaluate excess mortality in the city of Rio de Janeiro, Brazil, due to the COVID-19 pandemic (March 2020 to January 2022).

**Methods:**

Ecological study using secondary data from the Brazilian Mortality Information System, having the city of Rio de Janeiro as the unit of analysis. Excess mortality was estimated by the difference between the mean number of all expected deaths and the mean number of observed deaths, considering the 2015–2019 period. The quantile regression method was adjusted. The total value of cases above that expected by the historical series was estimated. Among all deaths, cases of COVID-19 and Influenza as underlying causes of death were selected. The ratio between excess mortality and deaths due to COVID-19 was calculated.

**Results:**

We identified an excess of 31,920 deaths by the mean (increase of 26.8%). The regression pointed to 31,363 excess deaths. We found 33,401 deaths from COVID-19 and 176 deaths from Influenza. The ratio between the verified excess mortality and deaths due to COVID-19 was 0.96 by the mean and 0.95 by the regression.

**Conclusion:**

The study pointed to large excess deaths during the COVID-19 pandemic in the city of Rio de Janeiro distributed in waves, including the period of the Influenza outbreak.

## RESUMO

**Objetivo:**

Analisar o excesso de óbitos no município do Rio de Janeiro (MRJ), RJ, durante a pandemia de COVID-19 (março de 2020 a janeiro de 2022).

**Métodos:**

Foi realizado um estudo ecológico com dados secundários do Sistema de Informação sobre Mortalidade cuja unidade de análise foi o MRJ. O excesso de mortalidade foi calculado pela diferença entre a média de óbitos esperados e a média dos óbitos observados levando-se em conta o período de 2015 a 2019. Foi ajustado um método de regressão quantílica. Calculou-se o valor total dos casos acima do esperado pela série histórica. Foram selecionados os óbitos por causa básica COVID-19 e *Influenza*. Também foi calculada a razão entre o excesso de óbitos e os óbitos atribuídos à COVID-19.

**Resultados:**

Foi identificado excesso de 31.920 óbitos pela média (26,8% de incremento). Pela regressão quantílica, encontrou-se excesso de 31.363 óbitos. Ocorreram 33.401 óbitos por COVID-19 e 176 por *Influenza*. A razão entre o excesso de óbitos encontrado e os óbitos atribuídos à COVID-19 foi de 0,96 pela média e 0,95 pela regressão quantílica.

**Conclusão:**

O estudo apontou grande excesso de óbitos durante a pandemia de COVID-19 no MRJ, distribuído em ondas, incluindo-se o período do surto de *Influenza*.

## INTRODUCTION

The new coronavirus (COVID-19) pandemic is the greatest health challenge of the 21^st^ century so far^1^. By August 2022, the World Health Organization (WHO) had confirmed around 577 million cases worldwide, with approximately 6.4 million deaths^2^. The American continent has the highest number of deaths. Therefore, it is the continent that has been most affected by the pandemic up to the present^2^.

Currently, Brazil ranks third in number of cases in the world (33.8 million cases), only behind the United States of America (USA) and India, and has an incidence rate of 161,001 cases per one million inhabitants^3^. The country also draws attention to the number of deaths, as it has the second highest number in the world, with 679 thousand deaths, only behind the USA, with a mortality rate of 3,229 deaths per one million inhabitants^2,3^.

The *Observatório COVID-19 BR*, an independent initiative whose objective is to disseminate quality information on COVID-19, estimates that for each registered death there must be between 1.21 and 1.41 deaths that have not yet been confirmed as due to COVID-19^4^. Estimating the degree of underreporting and the actual number of deaths from COVID-19 is one of the great challenges of public health in the world^5,6^, especially in developing countries, where the lethality of the disease is enhanced by limited access to healthcare services, the political scenario, and the higher incidence of the disease in the most vulnerable groups such as in Brazil^6–8^.

In this sense, assessing the magnitude of mortality from COVID-19 by measuring the excess deaths that occurred in a given period is an effective and increasingly used tool, as it enables to assess the consequences of the pandemic on health and mortality of the population^6,8,9^. The assessment of excess deaths allows estimating the direct and indirect effects of the pandemic on mortality, being an important tool for health management by identifying possible changes in the risk of death potentially associated with the pandemic^6,8,9^.

The distribution of deaths from COVID-19 in Brazil reflects the geographic, social, and economic heterogeneity of the country, with only five states accounting for 81% of occurrences: São Paulo, Rio de Janeiro, Ceará, Pernambuco, and Amazonas^10^. The disclosure of these deaths is directly related to the coverage of the Brazilian Mortality Information System (*Sistema de Informação sobre Mortalidade* – SIM) in these states, as well as the surveillance process of deaths from COVID-19^5^, whose purpose is to obtain clinical and epidemiological information on suspect cases of the disease, seeking to properly classify them and identify situations that may have contributed to the occurrence of the fatal outcome^11^.

The Brazilian Ministry of Health published the first guidelines for coding causes of death from COVID-19 on May 11, 2020, which facilitated the work of death investigators and standardized information to be entered into the SIM^12^. The temporal distribution pattern of total deaths in the municipality of Rio de Janeiro (MRJ), state of Rio de Janeiro, had slightly varied between 2012 and 2019, when, at the time of the first Chikungunya fever epidemic, there was excess deaths in relation to the means of the previous years^13^. With the advent of the COVID-19 pandemic, the absolute number of deaths from all causes significantly increased, from 60,438 in 2019 to 72,373 in 2020^14^.

An interesting fact that contributed to the reasonable number of deaths was the outbreak of Influenza A subtype H3N2, at a time when the reduction in cases of COVID-19 led to greater circulation of people^15^. Influenza circulation has been detected in many countries, including Brazil, and reported by the WHO^15^.

Taking this into consideration, the objective of this study was to analyze excess deaths during the COVID-19 pandemic, from the emergence of the wild-type Sars-CoV-2 virus, in March 2020, to the beginning of the circulation of the Omicron variant of concern, in January 2022, at MRJ, including the Influenza A H3N2 outbreak at the end of 2021.

## METHODS

An ecological study was carried out with secondary data whose unit of analysis was the MRJ. The data source was the SIM, and all deaths of MRJ residents that occurred between epidemiological weeks (EW) 1 of 2015 and 4 of 2022 for all causes of death were considered the study population. Data extraction took place on March 22, 2022.

The municipality is organized into 10 programmatic areas (PA), for the purpose of dividing public health actions, each with a General Coordination of Primary Health Care and a General Coordination of Emergency. The 10 PA comprise 162 neighborhoods with different characteristics, with a total estimated population of 6,775,561 inhabitants in 2022. Rio de Janeiro has known social inequalities due to the large number of people in extreme poverty, who live mostly in the city's 763 favelas and who have difficulty accessing quality healthcare services^16^.

Deaths due to COVID-19 infection (International Classification of Diseases — ICD-10 B34.2) and due to Influenza (ICD-10 J10 to J11) as the underlying causes were selected to verify the distribution over time and in relation to viruses and their variants. Data on respiratory viruses were obtained from laboratory surveillance systems (by the Laboratory Environment Management System [*Gerenciador de Ambiente Laboratorial*] – GAL) and sentinel influenza surveillance from the Superintendence of Health Surveillance (*Superintendência de Vigilância em Saúde* – SVS) of the Municipal Department of Health of Rio de Janeiro (*Secretaria Municipal de Saúde do Rio de Janeiro* – SMS-RJ). The dominant variant in the period was considered to be the one that had more than 50% of the viruses registered with the GAL.

The simple arithmetic mean of the number of deaths per EW was estimated from 2015 to 2019. The percentage change between means by EW was compared with the respective EW of 2020 and 2022. The same method was applied for sex, age group, and PA of residence. The ratio between the verified excess deaths and the deaths attributed to COVID-19 was calculated.

A quantile regression method^17,18^ was adjusted using monthly mortality rates from 2015 to 2019, with a tau value of 0.90, which generated a rate corresponding to the 90th quantile value for each month. These rates were subtracted from the general mortality rate observed during the study period, and with the sum of this excess, the total value of cases above the expected by the historical series was estimated. Subsequently, the ratio between the verified excess deaths and the deaths attributed to COVID-19 was calculated again. It was decided to present the quantile regression with monthly data to verify the adjustment of the model.

The analyses were performed using the R 3.6.1 software with the quantreg^18^ package and approved by the Research Ethics Committee of the SMS-RJ.

## RESULTS

**Figure 1:**
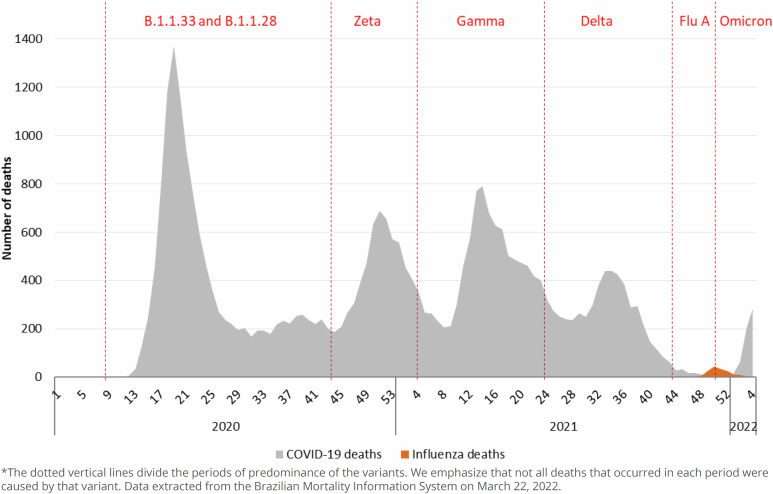
Distribution of deaths from COVID-19 and Influenza by epidemiological week, municipality of Rio de Janeiro (RJ), Brazil, 2020–2022*.

Source: SIM – SMS-RJ.

Since the beginning of the pandemic, in the EW 10 of 2020, until EW 4 of 2022, there were 33,401 deaths from COVID-19 and 176 deaths from Influenza. In Figure 1, we can observe the deaths and the distribution of the strains and variants of COVID-19 and the Influenza virus, according to the EW of the beginning of circulation in the MRJ. Regarding deaths, five peaks are well-marked by the predominance of the COVID-19 variants, and the small wave of Influenza can also be observed in the period between the predominance of the Delta variant and the Omicron variant (Figure 1).

By the mean of EWs from 2015 to 2019, 57,934 deaths of residents in the MRJ would be expected from EW 1 to EW 53 of 2020; 56,783 detahs from EW 1 to EW 52 of 2021; and 4,452 deaths from EW 1 to EW 4 of 2022, which totals 119,169 expected deaths from all causes in the studied period. However, we identified 151,089 deaths, which configured excess of 31,920 deaths or a global increase of around 26.8%. The ratio between the verified excess deaths and deaths attributed to COVID-19 was 0.96.

**Figure 2:**
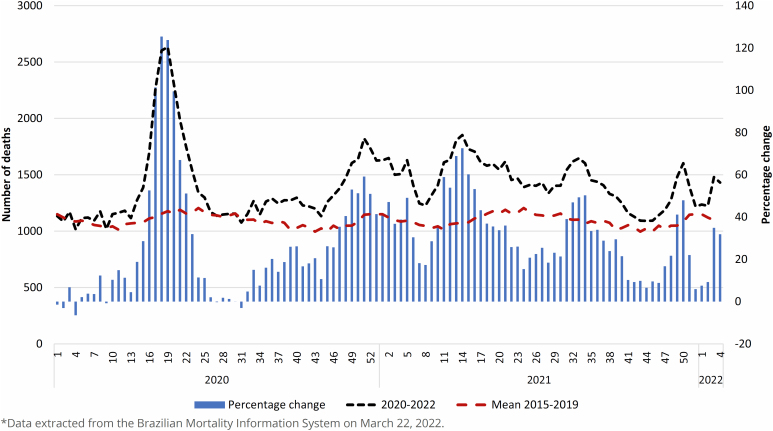
Mean deaths from 2015–2019, observed deaths from 2020–2022, and percentage change in deaths during the COVID-19 pandemic, municipality of Rio de Janeiro (RJ), Brazil, 2020–2022*.

Source: SIM – SMS-RJ.

In Figure 2, we can observe the mean number of deaths from 2015 to 2019, deaths from all causes that occurred in 2020-2022, and the percentage change by EW. The peak of 2020 occurred in EW 18, with an increase of 125.4% in deaths. The peak in 2021 occurred in EW 14, with a positive variation of 72.5%. In 2022, with the predominance of the Omicron variant, there was a new increase in deaths, with a positive variation of about 34% in EW 3.

The only period during the pandemic in which the percentage change in the number of deaths was minimal, with observed deaths approaching those expected, was between EW 26 and EW 32 of 2020. Subsequently, Zeta, Gamma, and Delta variants were detected. From EW 46 to EW 51 of 2021, the Influenza A H3N2 outbreak occurred, which again caused an increase in the number of deaths. When the observed number approached the expected number again in early 2022, the occurrence of the Omicron variant has probably contributed to more deaths. The vertical dotted lines reflect the beginning of virus isolation of a certain strain, which circulates at the same time as the previous strain(s), until it becomes dominant (Figure 1).

The percentage change by sex at the peak of 2020 was greater in men (142.3%) than in women (106.9%), with a smaller impact in 2021 for both sexes. Regarding age group, the greatest variation occurred between 40 and 69 years old, with an increase of 185.8% in 2020 and 119.7% in 2021. The 80-year-old age group is the one that most draws attention, as in 2020 it showed an increase of 102.7% in EW 19 and the peak of 2021 in EW 5 (68.4%), while the other age groups had their peaks in 2021 in EW 14, EW 19, and EW 21, according to Table 1. In the same table, distinct variations are verified between the PAs of residence in the two surveyed years.

**Table 1:** Epidemiological week with the highest percentage change in relation to the mean (2015–2019) by sex, age group (in years), and programmatic area, municipality of Rio de Janeiro (RJ), Brazil, 2020–2021*.

		EW 2020 peak	Variation %	EW 2021 peak	Variation %
MRJ		18	124.2	14	71.4
Sex					
	Women	19	106.7	14	70.6
	Men	18	142.3	14	72.2
Age group (years)					
	<40	19	39.3	19	39.1
	40–59	18	185.8	21	119.7
	60–79	18	152.9	14	107
	≥80	19	102.7	5	68.4
Programmatic area of residence					
	1.0	18	176.3	32	88.6
	2.1	18	129.7	32	64.4
	2.2	18	81.5	32	86.1
	3.1	18	174	15	63.7
	3.2	19	105.6	14	85.1
	3.3	18	134.3	14	75.3
	4.0	19	140.3	13	106.7
	5.1	19	139.8	14	85.7
	5.2	19	91	14	100.2
	5.3	18	143.2	16	107.8

* Data extracted from the Brazilian Mortality Information System on March 22, 2022. EW: epidemiological week; MRJ: municipality of Rio de Janeiro.

Source: SIM – SMS-RJ.

**Figure 3:**
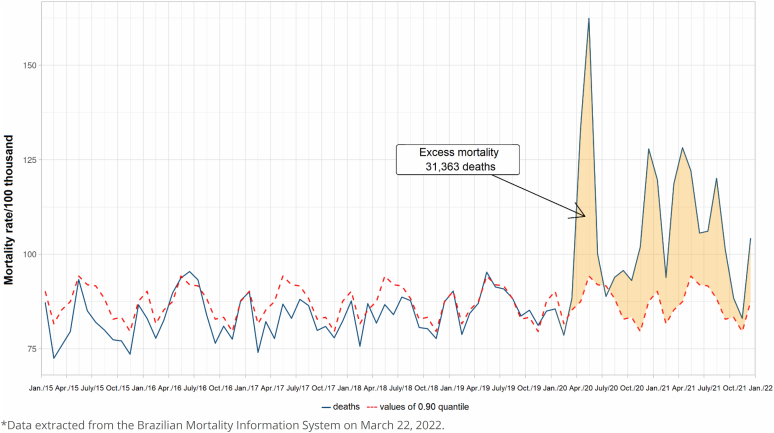
Expected and observed mortality rates obtained by quantile regression, municipality of Rio de Janeiro (RJ), Brazil, 2015–2022*.

Source: SIM – SMS-RJ.

In Figure 3, we present the observed and expected monthly general mortality rates between 2010 and 2021, generated by the quantile regression method. The sum of the excess of the difference between observed and expected deaths showed an excess of 31,363 deaths, attributable to the COVID-19 pandemic, slightly higher than the number estimated by the difference in relation to the mean. The ratio between the verified excess deaths and the deaths attributed to COVID-19 was 0.95 according to this method.

## DISCUSSION

The MRJ had excess deaths in the pandemic period of about 26.8% compared with the mean for the years 2015 to 2019. The model using quantile regression resulted in a similar value (26.2%). When working with national data in the first half of 2020^8^, Orellana et al. (2021) showed excess deaths of 112% in Manaus (state of Amazonas, Brazil) and 72% in Fortaleza (state of Ceará, Brazil), above the MRJ, which had 42%. In Ecuador, the percentage of excess deaths was 64% in 2020, and only 20% of these were attributed to COVID-19, mainly due to the low number of available tests^19^. In another study, researchers estimated an excess of 73% in Peru, the highest in the group of 77 countries studied^20^. In the USA, excesses of 36.5% were reported in April 2020 and 42.9% in January 2021^21^.

The number of deaths due to COVID-19 in the MRJ was greater than the excess due to the investigation of these deaths in accordance with the guidelines of the Brazilian Ministry of Health, when adopting laboratory, imaging, and clinical criteria^12^. The ratio between excess deaths and deaths from COVID-19 ranged from 0.96 using the mean method to 0.94 using quantile regression, possibly due to the investigation effort. In countries, such as France and Belgium, the ratio was well below 1, which suggested the inclusion of suspicious deaths as cases of COVID-19, as most countries presented the ratio above 1, which may reflect underreporting^20^. Men showed greater variation in the number of deaths than women in the MRJ at the peaks of 2020 and 2021. In descending order, the age groups 40–59, 60–79, and 80 years and over showed the greatest variations in peaks. The pattern was the same in Brazil in 2020^22^.

Variation was also noted between PAs, perhaps as a result of the heterogeneous spread of the disease throughout the city and the different characteristics of the areas. Due to the size of the MRJ, each PA behaves as large contiguous municipalities, which had pandemic peaks at different times^23^.

Deaths in the pandemic had a sudden increase in the first wave of COVID-19 from March 2020 onwards, with an accelerated need for hospital beds, especially specialized intensive care beds^5,6^. This had pressured healthcare services, which challenged managers and competed with other hospitalization, clinical, surgical, or trauma indications^5,6^. Even developed countries were affected and had a high number of deaths, such as the USA, Italy, the United Kingdom, and Spain, due to the emergence of a new coronavirus in susceptible populations^24^.

Deviations from the mean pattern of mortality can occur on a small scale, such as those caused by natural disasters (floods, landslides, hurricanes, and heat waves) or acute disease epidemics, such as the Chikungunya fever^13^ or the outbreak of Influenza A H3N2, as aforementioned, both at MRJ. The COVID-19 pandemic brings lethality associated with the disease to a great extent, whether caused by the infection itself or iatrogenic, by the inability to respond on the part of healthcare services, which are composed of physical infrastructure and human resources far below what is necessary^7^.

It is worth noting that, around EW 46 of 2021, when the pandemic seemed to be receding and the demobilization of hospital beds could be implemented, as well as the full return of health-related activities, the city of Rio de Janeiro was impacted by the emergence of Influenza A, with a new increase in demand for severe acute respiratory syndrome and intense search for primary health care. Again, the observed deaths, which were close to the expected ones, were distant from each other.

The start of vaccination against COVID-19, at the end of 2020, brought hope for the control of the pandemic in the world; however, the available vaccines are not capable of preventing the infection, although they drastically reduce hospitalization and deaths, even with variants such as the Omicron in circulation^25^. Population vaccination coverage, including the booster dose, seems to determine the number of deaths. In the MRJ, the population vaccination coverage with a complete two-dose schedule was above 80% between EW 51 and EW 52 of 2021 and the booster dose was slightly below 30%, at the time of the emergence of the Omicron variant^26^.

The discussion about preparing for other pandemics is imposed in the world. The WHO raises the issue of differences in the vaccination of the world's population as one of the obstacles to ending the current pandemic, as it allows the emergence of new variants, which can quickly spread. Thus, better structured healthcare services may not be the only need. A recent study on 177 countries identified that risk communication and community engagement are important for public health to achieve a better response^27^.

The response of the MRJ to the pandemic from 2021 onwards included the development of an Emergency Operations Center^28^. The organized structuring of public health allowed launching coordinated healthcare and surveillance actions, with the use of indicators to point out management directions^26^. Data on health care in the urgent and emergency network, hospitalization, and rapid testing in primary health care coupled with data from health surveillance information systems allow the implementation of a monitoring panel that helps in decision-making^26^.

The death control diagram, even in the simplest methodology according to the mean, can help inform when a public health event is moving beyond the mean of previous non-pandemic years. It is one more tool for management to understand their health and disease processes and propose new strategies in case of any unexpected change in the pattern. It has been used by management to monitor the city's health situation for several years. The control diagram of deaths from all causes can be incorporated into health surveillance indicators as strategic information.

This study has a limitation. There is a potential underreporting of deaths, already known in the literature when we analyze COVID-19. Although the Health Surveillance Divisions and the Coordination of Health Situation Analysis and of Epidemiological Surveillance of the SVS of SMS-RJ have made efforts to carry out investigations and draw conclusions regarding the deaths, there are still many open/undetermined cases, due to the great volume, especially in 2021.

We found important excess deaths during the COVID-19 pandemic in the MRJ, distributed in waves, and identified the outbreak of Influenza in the period. We recommend that new studies continue to monitor the evolution of deaths in the MRJ, seeking to collaborate with the creation of health policies that protect individuals from the same sociodemographic profiles, especially during new and future epidemics and/or pandemics that deviate from patterns already known in the literature.
